# Construction of *Escherichia coli* Whole-Cell Biosensors for Statin Efficacy and Production Test

**DOI:** 10.3389/fcell.2020.00404

**Published:** 2020-05-28

**Authors:** Huanjie Li, Qingda Wang, Rui Zhao, Yunshan Wang, Luying Xun, Huaiwei Liu

**Affiliations:** ^1^School of Medicine, Cheeloo College of Medicine, Shandong University, Jinan, China; ^2^State Key Laboratory of Microbial Technology, Shandong University, Qingdao, China; ^3^Medical Research and Laboratory Diagnostic Center, Jinan Central Hospital, Cheeloo College of Medicine, Shandong University, Jinan, China; ^4^School of Molecular Biosciences, Washington State University, Pullman, WA, United States

**Keywords:** synthetic biology, biosensor, statin, precision medicine, HMGR

## Abstract

Statins are widely used cholesterol-lowering drugs. Their potential application in anti-cancer treatment is also under investigation. The individual variance in statin response has been observed, which may be caused by the variation in human HMG-CoA reductase (hHMGR)—the inhibition target of statin drugs. Herein, we reported the design and construction of two *Escherichia coli* whole-cell biosensors. The first one is statin-efficacy testing sensor, which is composed of two separate modules: a hybrid mevalonate (MVA) pathway and a HMG-CoA sensing system. A truncated hHMGR was used as the key enzyme of the MVA pathway and a promiscuous transcription factor (TF) BsFapR was used as the HMG-CoA sensor. When hHMGR was inhibited by statins, HMG-CoA accumulated intracellularly and was sensed by BsFapR, which subsequently turned on its cognate promoter. This biosensor has the potential to be used as a “precision medicine” tool—selecting potent statin drugs for individual patients. The second one is a statin-production testing sensor, which is based on another promiscuous TF AraCM that can sense statins. This biosensor can be used in optimization of statin-producing strains. The prototypes of these two biosensors were successfully constructed and their further optimization is highly expected.

## Introduction

**FIGURE 1:**
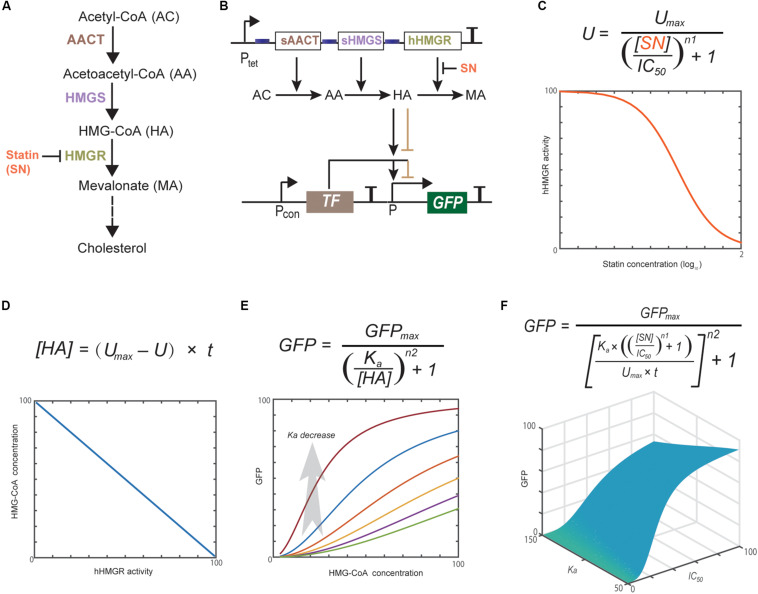
Statins block cholesterol synthesis through inhibiting hHMGR **(A)**. Schematic representation of the whole design of statin-efficacy testing sensor **(B)** and computational modeling of the dynamics of each component **(C–F)**. Modeling parameters are provided in Supplementary Material. Computational modeling indicates that the hHMGR activity, HMG-CoA accumulation, and GFP expression all show responses to statin in a dose-dependent mode.

Statins are a class of cholesterol-lowering drugs that are widely used in clinical practice (Rizzo et al., 2012; Garattini and Padula, 2017; Hennekens et al., 2017). They are competitive inhibitors of the HMG-CoA reductase (HMGR) that catalyzes the conversion of HMG-CoA to mevalonate (Endo, 1992; Istvan, 2003). HMGR is the rate-controlling enzyme of the mevalonate (MVA) pathway in human cell and is the target of statin inhibitors that regulate cholesterol concentration in human blood (Tabernero et al., 1999; Vögeli et al., 2019); hence, once HMGR is inhibited by statins, the cholesterol synthesis is impeded (Figure 1A). In addition to the usage as cholesterol-lowering medicines, recent studies also proposed the potential application of statins as anti-cancer drugs (Iannelli et al., 2018). Statins are able to affect cancer cell through mevalonate-dependent mechanisms (Mullen et al., 2016), and modulate specific signal transduction pathways to influence several cellular processes, such as angiogenesis, metastasis, apoptosis, and cell proliferation (Sopková et al., 2017). Despite the generally good treatment effect of statins, notable individual variation in response has been reported (Armitage, 2007; Wysockakapcinska et al., 2009; Sopková et al., 2017). This could be caused by the HMGR variance in different individuals. Human HMGR (hHMGR) has 1677 single nucleotide variants (NCBI), and diverse statin drugs are in the market as well; therefore, it is beneficial to construct a biosensor for pre-testing the inhibition effect of different statin drugs upon an individual hHMGR clinical treatment.

Natural statins are produced from polyketide biosynthetic pathways (PKS) of various microorganisms. For instance, lovastatin is produced by *Aspergillus terreus*, and mevastatin by *Penicillium citrinum* (Manzoni and Rollini, 2002). Tremendous efforts have been made to increase the production and yield of these strains, but their potentials have not been fully developed, mainly due to the complexity of PKS pathways and related enzymes (Campbell and Vederas, 2010). Although the biosynthesis of these compounds is not yet completely understood, rational metabolic engineering approaches targeting on construction of more efficient heterologous cell factories are on the way (Vajdić et al., 2014). For further optimization of either native or non-native statin-producing strains, development of biosensors that can quickly detect the statin production will be helpful. Until now, no such biosensor has been reported yet.

Signal-responsive transcription factors (TFs) play a major role in biosensor design (Mahr and Frunzke, 2016). The DNA binding activities of this type of TFs are affected by effectors, such as small molecules, ions, temperature, pH, or light, which subsequently lead to switch-like change of their cognate promoters (ON/OFF). These TFs can be directly applied in biosensor design (Siedler et al., 2014), or can be modified to construct non-natural biosensors (Bitzur et al., 2013; Morsut et al., 2016; Khatun et al., 2018). Herein, we reported the development of two prototypes of TF-based whole-cell biosensors. One is for statin-efficacy testing and another is for statin-production testing. For the statin-efficacy testing sensor, we used a HMG-CoA sensing TF to monitor the activity of HMGR. For the statin-production testing sensor, we employed a mutant TF that can directly sense the presence of statin. Both sensors were constructed in the *Escherichia coli* platform.

## Results and Discussion

### Design and Computational Modeling of the Statin-Efficacy Testing Sensor

Computational modeling has become a useful tool in developing synthetic gene networks with designed functions (Gould et al., 2014; Berset et al., 2017). Herein, we used it to guide the design and construction of the statin-efficacy testing sensor. The whole design consists of two separate modules: the hHMGR-based MVA pathway module and the MVA pathway monitoring module (Figures 1A,B). Statins inhibit the activity of hHMGR in the first module, which causes the accumulation of HMG-CoA. This process can be modeled with an IC_50_ equation (Figure 1C). A linear equation is used to model the relationship between hHMGR activity and HMG-CoA concentration (Figure 1D). In the second module, TF sensing the accumulation of HMG-CoA and turning on the expression of a reporter, GFP (Figure 1E). This process is modeled by a Hill equation (Figure 1F). Computational modeling indicates that the hHMGR activity, HMG-CoA accumulation, and GFP expression all show responses to statin in a dose-dependent mode (Supplementary Figure S1), suggesting they all can be indicators of statin efficacy.

A global equation was obtained by combining the three equations (Figure 1F). This equation indicated the *K*_a_ value, which represents the HMG-CoA concentration inducing half turn-on of the GFP promoter, is critical for the whole design. *K*_a_ value is mainly determined by the binding affinity of HMG-CoA sensing TF. If a TF with a too high *K*_a_ is used, GFP expression cannot be turned ON even when intracellular HMG-CoA accumulates to the maximum concentration caused the completely inhibition of hHMGR by statin. In this case, GFP expression will be constantly OFF no matter statin is present or not. If a TF with a too low *K*_a_ is used, GFP expression will be turned ON at low concentrations of HMG-CoA. In this case, a low concentration/efficacy of statin can lead to maximum GFP expression. Therefore, HMG-CoA-sensing TF is the key element that determines the performance of the statin biosensor.

### Expression of a Truncated hHMGR in *E. coli*

**FIGURE 2:**
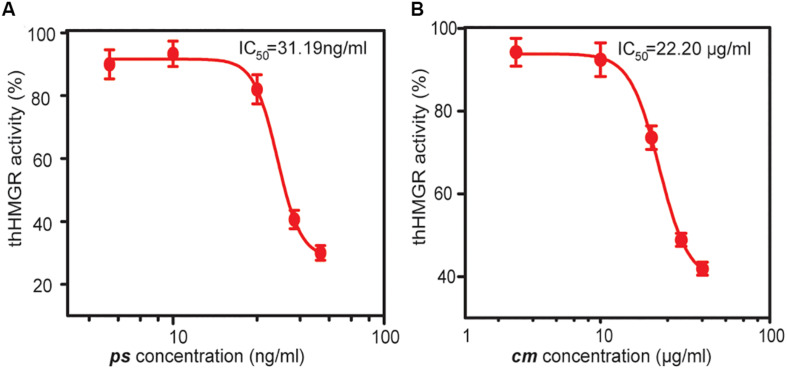
The inhibition effects of statin drugs on thHMGR. A pure simvastatin (*ps*) **(A)** and a commercial statin medicine (*cm*) **(B)** were used to treat purified thHMGR. The activity of untreated thHMGR was defined as 100%. The IC_50_ values of simvastatin and statin medicine were calculated to be 31.19 and 22.20 μg/mL, respectively.

The hHMGR (GenBank: XP_011541659.1) is a transmembrane protein that contains two main domains: a conserved N-terminal sterol-sensing domain anchored in the membrane of the endoplasmic reticulum, and a C-terminal catalytic domain in the cytosol, which is also the target domain of statins (Roitelman et al., 1992). We synthesized a codon optimized ORF encoding the C-terminal domain (residues 493-908) of hHMGR. This truncated hHMGR (thHMGR) gene was ligated to pET30a plasmid and transformed into *E. coli* BL21 (DE3). The N-terminal His-tag was used for purification. SDS-PAGE analysis indicated the expressed thHMGR was soluble in *E. coli* cytoplasm (Supplementary Figure S2A). After purification, we assayed the activity of thHMGR. The thHMGR catalyzes the reduction of HMG-CoA to mevalonic acid using NADPH as the coenzyme. We observed the NADPH consumption evidenced by the decrease of absorbance at 340 nm, indicating that the thHMGR expressed by *E. coli* retained the catalytic activity (Supplementary Figure S2B). To check the sensitivity of thHMGR to statin drugs, we added different concentrations of statins to the reaction system. The activity of thHMGR was significantly inhibited by both pure simvastatin (*ps*) and statin medicine (*cm*). The IC_50_ values were calculated to be 31.19 and 22.20 μg/mL, respectively (Figures 2A,B). The IC_50_ value of *ps* was three orders of magnitude lower than that of *cm*, which should be owing to *ps* is a relatively pure compound (simvastatin content ≥97%), while *cm* is clinical used drug containing much less simvastatin.

### Construction of the MVA Pathway With thHMGR

**FIGURE 3:**
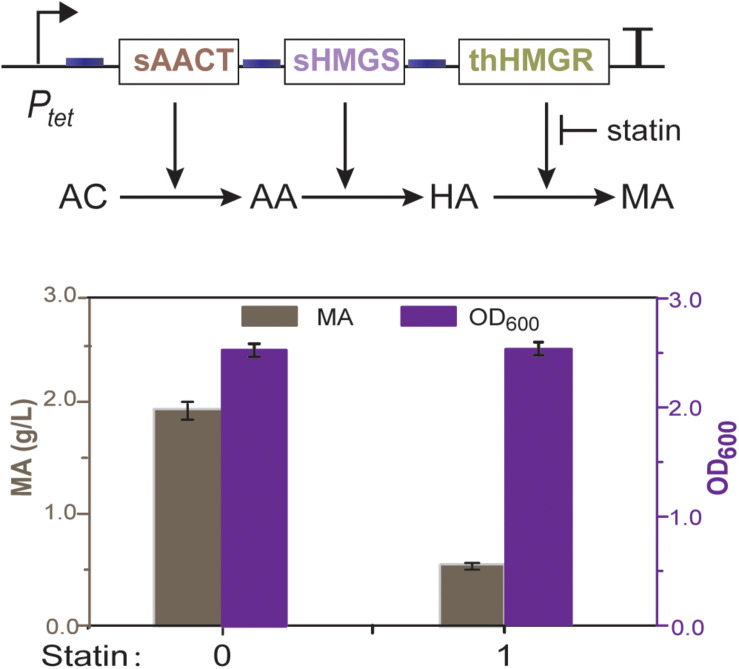
Mevalonate production of the thHMGR-based MVA pathway. *E. coli* BL21 harboring the MVA pathway was used. Pure simvastatin (50 ng/mL) was added into the LB medium and 1.9 g/L of mevalonic acid was produced.

To check whether *E. coli* has unspecific HMGR activity that may disturb further analysis, we first constructed an operon encoding acetyl-CoA thiolase (AACT) and HMG-CoA synthase (HMGS). These two enzymes are obtained from *Saccharomyces cerevisiae*, which can convert acetyl-CoA to HMG-CoA (Figure 1B). This operon was driven by an aTc (anhydrotetracycline) inducible promoter P_tet_ (P_tet_-AACT-HMGS) and expressed in *E. coli* BL21. HPLC analysis showed that no mevalonic acid was produced after aTc induction, indicating no unspecific HMGR activity is present in *E. coli*. We then co-expressed thHMGR with AACT and HMGS as one operon, using the same promoter P_tet_ (P_tet_-AACT-HMGS-thHMGR). HPLC analysis showed that when aTc was added, 1.9 g/L of mevalonic acid was produced (Figure 3). These results indicated the thHMGR-dependent MVA pathway was successfully constructed in *E. coli*. To test the inhibition effects of statin drugs at whole cell background, we added *ps* (50 ng/mL) simultaneously with aTc. The production of mevalonic acid was decreased to 0.6 g/L, indicating statin can penetrate *E. coli* cell and inhibit the intracellular thHMGR enzyme.

### Construction of TF-Based Biosensor for Detecting HMG-CoA

**FIGURE 4:**
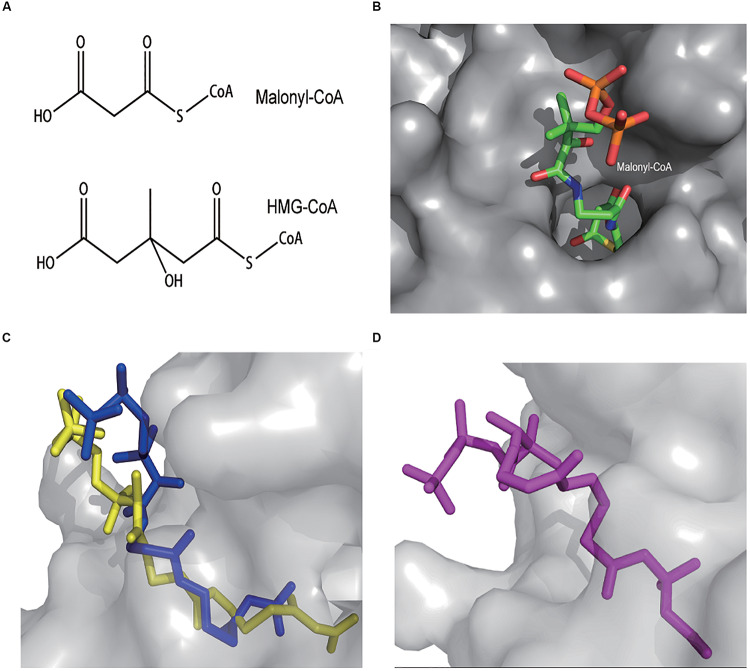
Both malonyl-CoA and HMG-CoA have a carboxyl group and a carbonyl group **(A)**. The FapR dimer forms a “hot-dog” pocket where malonyl-CoA locates in (top view). The malonyl moiety is deeper inside whereas the CoA moiety is mostly outside **(B)**. AutoDocK simulation of BsFapR-malonyl-CoA interaction (side view). Dark blue represents the actual binding conformation determined by crystallography and light yellow represents the simulated binding conformation **(C)**. AutoDocK simulation of BsFapR-HMG-CoA interaction **(D)**.

No HMG-CoA specific TF has been reported so far. We turned to the TFs that can sense analogs of HMG-CoA. Three types of fatty acyl-CoA binding TF have been identified (Albanesi et al., 2013). FadR that binds long-chain fatty acyl-CoAs, DesT that binds unsaturated acyl-CoAs, and FapR that binds malonyl-CoA. Among them, FapR attracted our attention due to its ligand malonyl-CoA is structurally the most similar to HMG-CoA (Figure 4A). The FapR-ligand binding mechanism has been elucidated at structural level (Albanesi et al., 2013). Briefly, the ligand binding domain of FapR forms a “hot-dog” fold (Figure 4B). The malonyl moiety is involved in the binding whereas the CoA moiety locates outsides of the binding cavity. The binding of malonyl-CoA leads to series of conformational changes of FapR and finally makes it release from its DNA binding site. Hence, FapR is a derepression TF and malonyl-CoA is its native inducer.

To predict the possibility of FapR-HMG-CoA binding, we performed protein-ligand binding simulations using the AutoDock software. The *Bacillus subtilis* FapR (BsFapR, PDB: 2F3X) was used as the docking protein. The BsFapR-malonyl-CoA binding simulation gave a result very similar to the experimental confirmed conformation (Figure 4C), suggesting the algorithm and parameters used for BsFapR simulation was reliable. The BsFapR-HMG-CoA binding simulation produced a result having similar binding energy but higher affinity to that of BsFapR-malonyl-CoA (Figure 4D and Supplementary Table S1). These results suggested HMG-CoA can be a candidate ligand of BsFapR.

**FIGURE 5:**
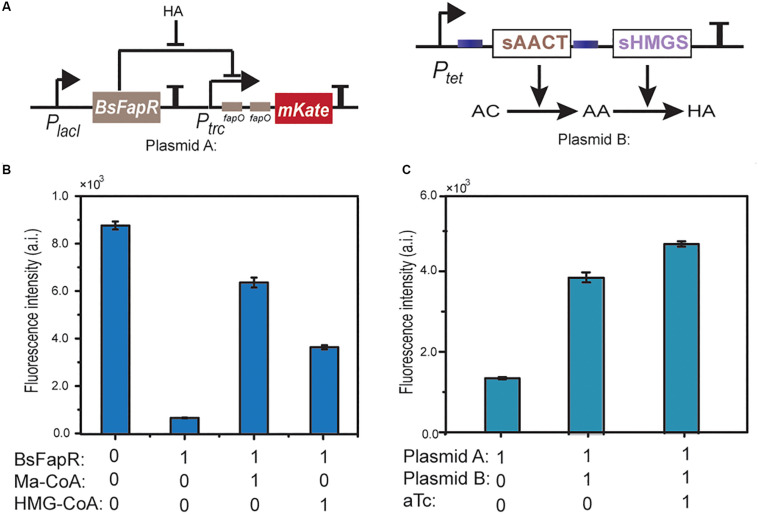
Schematic representation of the HMG-CoA (HA) sensing module and the HMG-CoA producing module **(A)**. *In vitro* transcription-translation test of the derepression effect of HMG-CoA. A DNA fragment containing *P*_trc_-(*fapO*)_2_-mKate was used as the template. Ma-CoA represents malonyl-CoA. The number 0 represents no addition and 1 represents addition **(B)**. *In vivo* test of the derepression effect of HMG-CoA. *E. coli* BL21 containing plasmid A or both A and B plasmids were used. aTc is the inducer of *P*_tet_ **(C)**.

Both *in vitro* and *in vivo* experiments were performed to test the actual interaction between BsFapR and HMG-CoA. For *in vitro* experiments, BsFapR was expressed in *E. coli* BL21 (DE3) and purified using the N-terminal His-tag. A reporter DNA fragment was constructed, which contains an artificial promoter composed of *P*_trc_ core sequence (−10 and −35) and two FapR binding sites (*fapO*). After this promoter is the mKate reporter gene (Figure 5A). The purified BsFapR and constructed reporter DNA fragment were mixed in the *in vitro* transcription-translation system. If HMG-CoA can actually work as a ligand of FapR, it will induce the release of FapR from the fapO site, transcription of mKate mRNA and subsequent translation will be detected from the *in vitro* system. Results showed that high amount of mKate was synthesized when no BsFapR was present, whereas much lower amount was synthesized when BsFapR was added into the *in vitro* system. When HMG-CoA was added simultaneously with BsFapR, the amount of synthesized mKate significantly increased. As a control, when malonyl-CoA was added simultaneously with BsFapR, the amount of synthesized mKate was higher than that of HMG-CoA addition (Figure 5B).

For the *in vivo* experiments, a plasmid was constructed containing the reporter DNA. When the reporter plasmid was transformed into *E. coli* BL21, no obvious mKate expression was detected. However, when it was co-transformed with P_tet_-AACT-HMGS, the HMG-CoA producing plasmid, high expression of mKate was observed. The expression could be further increased when aTc was added (Figure 5C). Both *in vitro* and *in vivo* results indicated that in consistent with AutoDock simulation, HMG-CoA indeed, although not as efficient as malynol-CoA, can react with BsFapR and induce the derepression process.

### Construction of the Statin-Efficacy Testing Sensor

**FIGURE 6:**
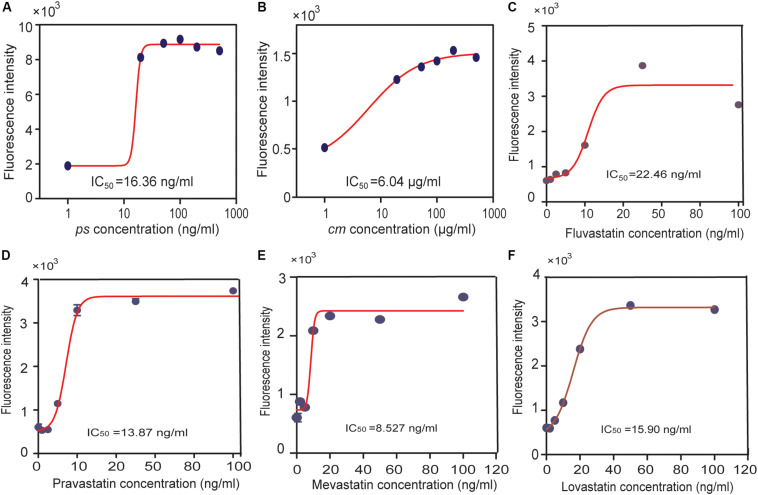
Using the statin-efficacy testing sensor in drug test. *E. coli* BL21 harboring both the reporter and the P_tet_-AACT-HMGS-thHMGR plasmids were used. A pure simvastatin (*ps*), **(A)** a commercial simvastatin containing drug (*cm*), **(B)** and other statins were tested including fluvastatin **(C)**, pravastatin **(D)**, mevastatin **(E)**, lovastatin **(F)**. IC_50_ values of different statins were all tested by this sensor and calculated.

The reporter and the P_tet_-AACT-HMGS-thHMGR plasmids were co-transformed into BL21 to make the final whole-cell biosensor. Pure simvastatin (*ps*) was firstly tested on the obtained biosensor. Without presence of *ps*, mKate expression was low, indicating thHMGR was active. When *ps* was added (20, 50, 100, 200, 500 ng/mL), mKate expression was turned on, indicating thHMGR was inhibited by *ps*. The IC_50_ value of *ps* was calculated to be 16.36 ng/mL using the mKate fluorescence intensity as the indicator (Figure 6A). The commercial medicine (*cm*) were also tested (5, 10, 20, 50, 100 μg/mL) on this biosensor and its IC_50_ was calculated to be 6.04 μg/mL (Figure 6B). The IC_50_ values calculated from mKate intensity were about half to one third of the data calculated from the enzyme inhibition experiments (Figure 2). This should be caused by the relative lower sensitivity BsFapR (*K*_a_) to HMG-CoA. Other statins with high-purity (≥97%), including lovastatin, mevastatin, pravastatin, fluvastatin were also tested by this sensor and their IC_50_ values were calculated (Figures 6C–F). We also performed toxicity testing experiments with these statins. None of them showed inhibition effect on growth of *E. coli* BL21 (Supplementary Figure S3), indicating statins only target for thHMGR in the strain.

It needs to emphasize that the biosensor reported herein is the first version. Although it successfully indicates the statin efficacy difference, there is still much room for its improvement. First, the MVA pathway module needs to be further optimized. The range of mevalonate production is between 0.6 g/L (with excess statin) to 1.9 g/L (without statin), HMG-CoA production should be also in a narrow range, which limits the sensitivity of the biosensor. Using more efficient AACT and/or HMGS enzymes to enhance the metabolic flux to MVA pathway, or balance their activities should further amplify the HMG-CoA production range, hence improve the sensitivity of the sensor. Second, the binding affinity of BsFapR to HMG-CoA is critical for the statin sensor. Using protein engineering methods to construct FapR mutant with higher HMG-CoA affinity should significantly increase the sensitivity of biosensor.

### Construction of the Statin-Production Testing Sensor

**FIGURE 7:**
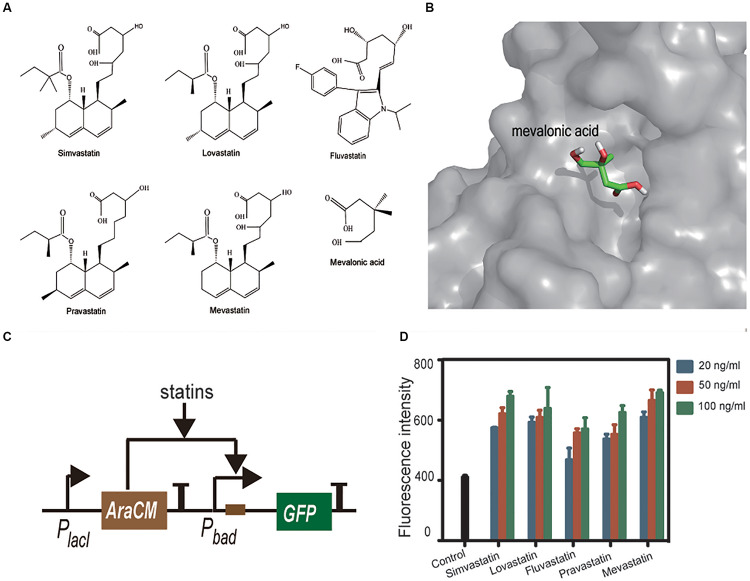
Statins **1** and mevalonic acid **2** in linear form **(A)**. AutoDocK simulation of AraCM-mevalonic acid interaction **(B)**. Schematic presentation of the statin sensing circuit **(C)**. Statins were tested in *E. coli* BL21 containing the statin-sensing plasmid. Control is the strain without statin treatment. All statins induced more or less expression of GFP, and dose-response relationships were also observed **(D)**.

Compared with the statin-efficacy testing sensor, the design principle and construction of statin-production testing sensor is relatively simpler. Statins are extracellular products of some microorganisms; hence, a co-cultured strain containing a statin-sensing TF and a reporter gene can be used as a whole-cell biosensor. No such TF has been reported so far. Istvan and Deisenhofer (2001) reported that statins interact with HMGR through the carboxyl and hydroxyl groups on their tails, and the binding pocket of HMGR locates at an open zone of surface. This finding gave us a clue that the TF having a similar pocket and statin tail-like ligand may have the potential to bind statins. AraCM is a mutant of AraC (the arabinose sensing TF) that can sense mevalonic acid, whose structure is very similar to that of statin tails (Figure 7A). The crystal structure of AraCM is not available. We modeled its 3D structure by using SWISS-MODEL. AraC (PDB: 1XJA) at 2.4 Å resolution was used as the template (61% sequence similarity). The global QMEAN score is 0.65 for this model, implying the predicted models are valid. The AraCM-mevalonic acid binding simulation was performed using the AutoDock software. The simulation results showed that mevalonic acid binding site locates at a pocket of AraCM surface (Figure 7B).

Based on this finding, we constructed a statin-sensing plasmid, which contains AraCM, its cognate promoter P_bad_, and the GFP reporting gene (Figure 7C). The plasmid was transformed into *E. coli* BL21. Pure compounds of simvastatin, lovastatin, fluvastatin, pravastatin, and mevastatin were used to treat the plasmid containing *E. coli*. All statins induced more or less expression of GFP, and dose-response relationships were also observed (Figure 7D). These results indicated that AraCM indeed can sense statins and the statin-sensing biosensor was successfully constructed. The AraCM-based sensor can be introduced into statin-producing strains directly, or the *E. coli* containing AraCM-based sensor can be co-cultured with statin-producing strains for screening more efficient statin-producing cell factories. It is noteworthy that the increase of GFP expression caused by statins were less than twofold, indicating the sensitivity of AraCM to statins is limited. To construct more sensitive biosensors, further screening more statin-sensitive TFs or modifying AraCM with protein engineering methods is required.

## Conclusion

In conclusion, we successfully developed two prototypes of statin biosensors. The first one has the potential to be used as a tool for “precision medicine.” Among the 1677 single NCBI of hHMGR, 176 are missense variants and 11 are frame-shift variants, which alters the amino acid sequence of hHMGR. Statins are one of the most commonly used medications to lower cholesterol (Ahmed et al., 2020), which have been known to cause various side effects such as muscle pain, muscle weakness, rhabdomyolysis, especially high-dosage statins (Bitzur et al., 2013; Ahmed et al., 2020; Won et al., 2020). By replacing the thHMGR gene with the individual’s variant in the statin-efficacy testing sensor, we can test which statin drug has the best inhibition effect on his/her HMGR activity, and what is the best dosage. Based on this test, we can provide personal tailored therapy plan, and hence it is possible to control the usage of statins and avoid side effect of the drugs, such targeting a drug(s) to an individual patient or a subclass of patients is a strategy of precision medicine. The second biosensor can be used for screening of statin-producing strains. The AraCM based reporter also can be transferred into statin-producing strains, which may work as a useful tool for strain optimization or metabolic engineering modification.

There are still some weaknesses in application of sensors, such as the human-derived proteins being expressed in *E. coli* requiring future optimization, as well as solubility problems of statins during the experiment. These issues may be the limiting aspects of sensor applications. Further optimization of these two biosensors with more rounds of “design-construction-test-learn” cycles is expected.

## Materials and Methods

### Strains and Growth Conditions

*Escherichia coli* DH5α was used as the plasmid construction host, *E. coli* BL21 (DE3) was used as the protein expression host and the biosensor construction platform. Luria-Bertani (LB) medium (1% NaCl, 0.5% yeast extract, and 1% tryptone) was used for *E. coli* cultivation. When needed, antibiotics were added at required concentration. Simvastatin (*ps*) was purchased from Sigma-Aldrich, commercial simvastatin containing drug (*cm*) was purchased from a local pharmacy, lovastatin, mevastatin, pravastatin, and fluvastatin were purchased from MedChem Express.

### Plasmid Construction and DNA Manipulation

Plasmids used in this study are listed in Table 1 and primers are listed in Supplementary Table S2. Plasmid construction was performed using the In-Fusion method (Invitrogen). The ORF of thHMGR sequence was synthesized by the BGI Company and its codon was adapted to fit the *E. coli* translation system. The ORF of BsFapR was cloned from *B. subtilis* and the ORF of AraC was cloned from *E. coli* DH5α. Site-specific mutations were performed on AraC to make AraCM using a previously reported method (Roitelman et al., 1992).

**TABLE 1:** Strains and plasmids used in this study.

**Plasmids/strains**	**Genetic characteristics**	**Source**
**Plasmids**		
P_Trc_-His2A	P_Trc_ promotor plasmid, Amp^R^	Lab collection
pkd46	P_BAD_ promotor carrying *araC* gene, Amp^R^	Lab collection
pET30a	enzymes expression plasmid, Kan^R^	Lab collection
pBAD33MevT	P_BAD_ promotor carrying *araC* gene, Cm^R^	addgene#17814
pLeiss-3a-mkate	pLeiss plasmid with *rfp* report gene, Amp^R^	Lab collection
pTDFOR-sfGFP	pTD plasmid with *gfp* report gene, Amp*^R^*	Lab collection
P_Trc_-bFapR	p_Trc_ plasmid with *bfapR* regulator, Amp^R^	Lab collection
pET-HMGR	pET carrying HMGR protein	This study
pET-bFapR	pET carrying bFapR protein	This study
P_Trc_-fapo-mkate-T7-bFapR	P_Trc_ carrying *fapo* site, T7 promotor, *fapR*, *mkate* genes	This study
P_Trc_-fapo-mkate-lacI-bFapR	P_Trc_ carrying *fapo* site, *lacI* promotor, *fapR*, *mkate* genes	This study
P_Trc_-(fapo)_2_-mkate-lacI-bFapR	P_Trc_ carrying two *fapo* sites, *lacI* promotor, *fapR*, *mkate* genes	This study
P_Trc_-His2A-araCM	P_Trc_ carrying *araC* mutant gene	This study
P_Trc_-His2A-araC-Mev-pBad-GFP	P_Trc_ carrying *araC* mutant gene, pBad promotor, and *gfp* genes	This study
P_Trc_-His2A-T7-araC-Mev-pBad-GFP	P_Trc_ carrying araC mutant gene, T7 promotor, pBad promotor, and GFP	This study
P_Tet_-AACT-HMGS	P_Tet_ plasmid carrying AACT and HMGS gene	This study
P_Tet_-AACT-HMGS-thHMGR	P_Tet_ plasmid carrying AACT, HMGS and HMGR gene	This study
***E. coli* strains**		
BL21(DE3)	Host strain for enzymes expression	Lab collection
BL21-P_Trc_-bFapR-mkate	BL21 (DE3) with P_Trc_-fapo-mkate-lacI-bFapR	This study
BL21-P_Tet_-HMGR-P_Trc_-bFapR-mkate	BL21 (DE3) with P_Trc_-fapo-mkate-lacI-bFapR and pTet-AACT-HMGS-HMGR	This study
BL21-P_Trc_-T7-araC-P_Bad_-GFP	BL21 (DE3) with P_Trc_-His2A-T7-araC-Mev-pBad-GFP	This study
BL21-P_Tet_-HMGR-P_Trc_-T8-araC-pBad-GFP	BL21 (DE3) with P_Trc_-His2A-T7-araC-Mev-pBad-GFP and P_Tet_-AACT-HMGS	This study
BL21-P_Tet_-AACT-P_Trc_-bFapR-mkate	BL21 (DE3) with P_Trc_-fapo-mkate-lacI-bFapR and P_Tet_-AACT-HMGS-HMGR	This study
BL21-P_Tet_-AACT-P_Trc_-T8-araC-pBad-GFP	BL21 (DE3) with P_Trc_-His2A-T7-araC-Mev-pBad-GFP and P_Tet_-AACT-HMGS	This study
BL21-pET-HMGR	BL21 (DE3) with pET-HMGR	This study
BL21-pET-bFapR	BL21 (DE3) with pET-bFapR	This study

### Protein Expression and Purification

The protein ORFs were cloned into vector pET30a at the *Bam*HI and *Xho*I sites with an N-terminal His-tag. The recombinant plasmids were transformed into *E. coli* BL21 (DE3). The recombinant *E. coli* was grown in LB at 30°C with shaking until OD_600 nm_ reached about 0.6. When isopropyl-β-D-thiogalactopyranoside (IPTG) was added, the final concentration was 0.2 mM, and the cells were further cultivated at 16°C for 20 h. Cells were harvested via centrifugation, washed twice with ice-cold lysis buffer (50 mM NaH_2_PO_4_, 300 mM NaCl, and 20 mM imidazole, pH 8.0), and broken through the high pressure crusher SPCH-18 (STANSTED). Cells were removed via centrifugation and the supernatant was loaded onto the nickel-nitrilotriacetic acid (Ni-NTA) agarose resin (Invitrogen). The resin was washed with 5 column volumes (CV) of resuspension buffer, and added the His-tagged protein with an elution buffer (50 mM NaH_2_PO_4_, 300 mM NaCl, and 250 mM imidazole, pH 8.0). The eluted fractions with the c were loaded onto a PD-10 desalting column (GE Healthcare) for buffer exchange (25 mM Hepes, 300 mM NaCl, and 10% glycerol, pH 8.0). Dithiothreitol (1 mM) was added into the resuspension buffer. Purity of the proteins was analyzed by SDS-PAGE.

### HMG-CoA Reductase Activity Assay

The activity of thHMGR was assayed using the HMGR-CoA Reductase Assay Kit (Sigma CS1090). Experiments were performed by following the manufacturer’s protocol. Briefly, the reaction was conducted in a 96-well UV plate and monitored with a microplate reader (Synergy H1). Purified thHMGR, HMG-CoA, and the coenzyme NADPH were mixed in the reaction buffer. The total volume of the reaction mixture was 100 μL. After a 10 s blending, the 340 nm absorbance of the mixture was continuously monitored for 10 min. The HMGR activity was normalized to the protein concentration. For statin inhibition efficacy assay, different amount of statin drugs were added into the reaction mixture. The thHMGR activity data were plotted against the statin concentration data, and the IC_50_ was calculated by fitting the curve into an IC_50_ equation embedded in Graphpad.

### HPLC Analysis

For mevalonate production analysis, 50 μl overnight cultures of *E. coli* BL21/P_tet_-AACT-HMGS and *E. coli* BL21/P_tet_ -AACT-HMGS-thHMGR were individually inoculated into 5 mL fresh LB medium and cultivated at 30°C with 200 rpm shaking. After 2 h, 50 ng/mL of anhydrotetracycline (aTc, Clontech) was added and the cultivation continued for 3 h. After removing the cells by centrifugation, the cultivation supernatant was treated with 0.5 mM of sulfuric acid and then subjected to HPLC analysis. Shimadzu HPLC equipped with a Bio-Rad HPX-87H column (Bio-Rad) and a RID-10A refractive index detector were used. The HPX-87H column was kept at 55°C. Sulfuric acid solution (0.5 mM) was used as mobile phase and the flow rate was set to 0.4 mL/min; mevalonic acid lithium (purity ≥97%) was used as the external standard for quantificational analysis.

### *In vitro* Transcription-Translation Assay

The *in vitro* protein synthesis kit PURExpress^®^, RNA polymerase, and RNAase inhibitor were purchased from New England Biolabs. Purified BsFapR and the DNA fragment containing P_Trc_-(fapO)_2_-mKate were used. The experiment was carried out according to the manufacturer’s instructions with slight adjustments. Briefly, The reaction system contained 5 μL solution A, 3.5 μL solution B, 10 units RNase inhibitor (NEB), 100 ng DNA template (0.2 pmol), and 0.8 μL *E. coli* RNA polymerase. When required, 0.2 μL FapR (0.6 pmol) and 0.8 μL Malonyl-CoA (30 pmol) or HMG-CoA (60 pmol) was added. The total volume of the mixture was 12.5 μL. The reaction mixture was incubated at 37°C for 3 h, followed by cooling to 4°C.

To check the synthesized mKate, the reaction mixture was diluted, then subjected to fluorescence analysis. The microplate reader (Synergy H1) was used and the excitation and emission wavelengths were set to 588 and 633 nm, respectively.

### *In silico* Analysis

Computational modeling was performed using MATLAB (v2014b). The BsFapR-ligands and AraCM-mevalonic acid interactions were performed using AutoDock (v4.2.6) (Albanesi et al., 2013). The BsFapR structural data was from Protein Database Bank (PDB: 2F3X). Its docking center was assigned with *x* = 4, *y* = −28, and *z* = 60. Search space was assigned with *x* = 30 Å, *y* = 60 Å, and *z* = 50 Å, and exhaustiveness was assigned with 10. The 3D structure of AraCM was generated by SWISS-MODEL^1^. AraC (PDB: 1XJA) at 2.4 Å resolution was used as the template (61% sequence similarity). The generated model was analyzed using PyMOL-1.5.0.3. Its docking center was assigned with *x* = 122.1, *y* = 28.9, and *z* = 0.722. Search space was assigned with *x* = 44 Å, *y* = 44 Å, and *z* = 32 Å, and exhaustiveness was assigned with 10. Grid maps were automatically computed.

### Using Whole-Cell Biosensors in Drug Tests

*Escherichia coli* whole-cell biosensors were grown and tested in LB medium. Overnight cultured bacteria (100 μL) was inoculated into 10 mL fresh LB in 50 mL corning tubes and cultivated at 37°C for 3 h with 200 rpm shaking. Then statin drugs and inducer (aTc, 0.1 μg/mL) were added simultaneously into the culture. The cultivation was continued for another 2 h, then 1mL of *E. coli* cells were collected by centrifugation and washed twice with 0.9% NaCl solution. The obtained cells were re-suspended in 200 μL of 0.9% NaCl solution and subjected to fluorescence analysis. The microplate reader (Synergy H1) was used and for mKate analysis, the excitation and emission wavelengths were set to 588 and 633 nm, respectively; for GFP analysis, the excitation and emission wavelengths were set to 485 and 528 nm, respectively. Cell density (OD_600 nm_) was also analyzed by the microplate reader and the fluorescence intensity was normalized to per unit OD_600 nm_ of cells.

## Data Availability Statement

The raw data supporting the conclusions of this article will be made available by the authors, without undue reservation, to any qualified researcher.

## Author Contributions

LX and HWL conceived and designed the study. HJL and QW performed the experiments. RZ performed the computational work. HWL drafted the manuscript. All authors have given approval to the final version of the manuscript.

## Conflict of Interest

The authors declare that the research was conducted in the absence of any commercial or financial relationships that could be construed as a potential conflict of interest.

## Footnotes

1 http://swissmodel.expasy.org/
