# Supplementary material for: Construction of Escherichia coli Whole-Cell Biosensors for Statin Efficacy and Production Test
Source: Front Cell Dev Biol. 2020 May 28;8:404. doi: 10.3389/fcell.2020.00404 (PMC7326143; doi:10.3389/fcell.2020.00404)
Supplement: Supplementary file 1 [file Data_Sheet_1.pdf]

Supplementary Material

Huanjie Li[1,2,3], Qingda Wang[2], Rui Zhao[2], Yunshan Wang[1,3], Luying Xun[2,4], Huaiwei Liu[2,*]

[1]School of Medicine, Cheeloo College of Medicine, Shandong University, Jinan, Shandong, 250012, People's Republic of China.

[2]State Key Laboratory of Microbial Technology, Shandong University, Qingdao, Shandong, 266200, People's Republic of China.

[3]Medical Research & Laboratory Diagnostic Center, Jinan Central Hospital, Cheeloo College of Medicine, Shandong University, Jinan, Shandong, 250013, People's Republic of China.

[4] School of Molecular Biosciences, Washington State University, Pullman, WA, 99164-7520, USA.

Corresponding Author

*Huaiwei Liu, Tel: +86 532 5863 1572. E-mail: liuhuaiwei@sdu.edu.cn;

Table of Contents

Supporting methods 2

Parameter values used for computational modeling in Figure 1F 2

Supporting Figures 3

Figure S1. Computational modeling indicates that the activity of thHMGR, HMG-CoA concentration, and GFP all relate to statin concentration. 3

Figure S2. SDS-PAGE analysis of the thHMGR expression in *E. coli* (A) and activity assay of the purified enzyme. S1–S3 are three repetations (B). 4

Figure S3. Toxicity test of statins on *E. coli* BL21. 5

Supporting Tables 6

Table S1 Docking results of ligands in BsFapR 6

Table S2 Primers used in this study 6

Supporting methods

Parameter values used for computational modeling in Figure 1F

Parameter	Value	Unit
U_{max}	100	mg/L/h
[SN]	0–100	ng/mL
IC_{50}	1-100	ng/mL
t	1	h
K_a	1-100	mg/L
n1	2	dimensionless
n2	2	dimensionless
U_{max}	100	arbitrary unit

Supporting Figures

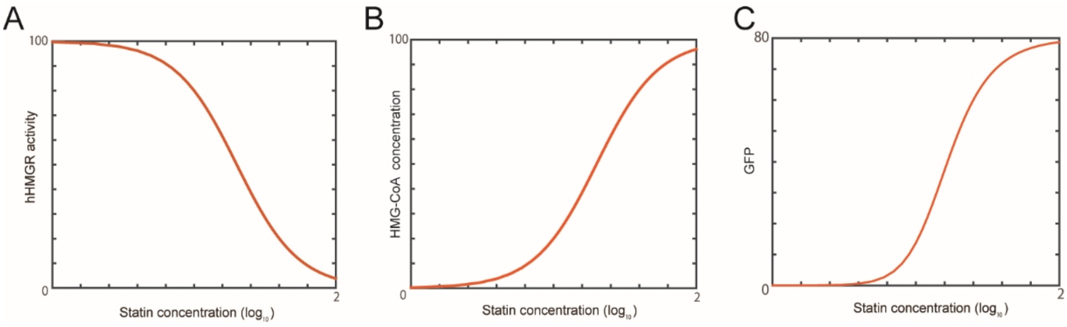

Figure S1. Computational modeling indicates that the activity of thHMGR, HMG-CoA concentration, and GFP expression all relate to statin concentration. They show responses to statin in a dose-dependent mode, suggesting they all can be indicators of statin efficacy.

Parameter values used:

Parameter	Value	Unit
U_{max}	100	mg/L/h
[SN]	0–100	ng/mL
IC_{50}	50	ng/mL
t	1	h
K_a	50	mg/L
n1	2	dimensionless
n2	2	dimensionless
GFP_{max}	100	arbitrary unit

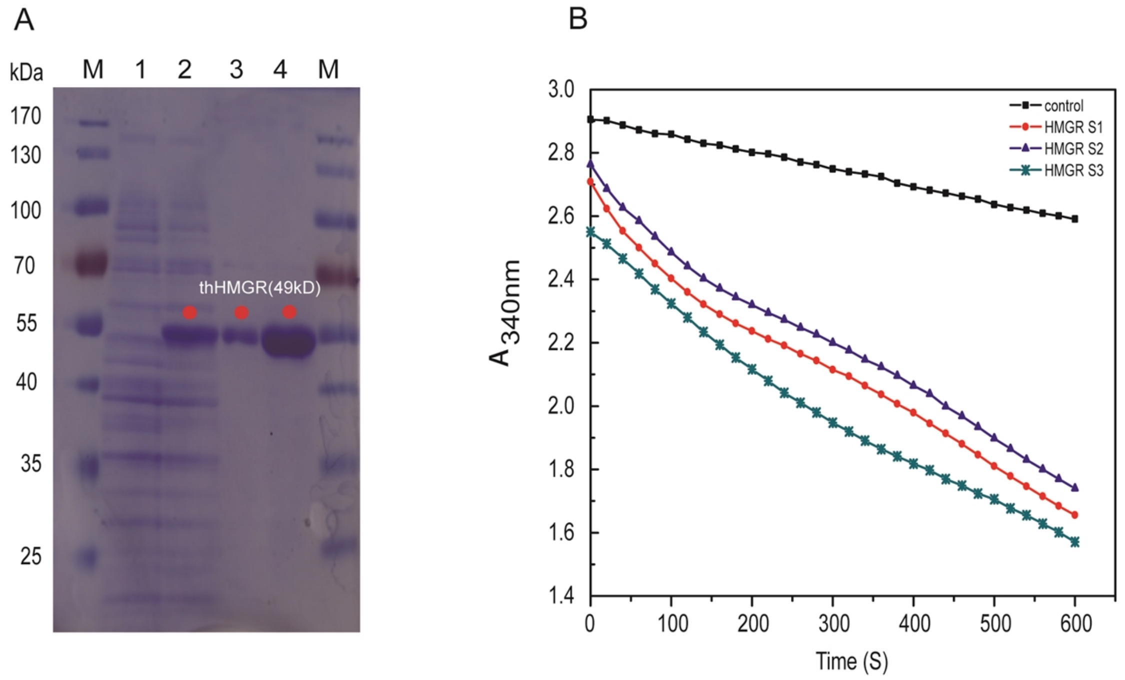

Figure S2. SDS-PAGE analysis of the thHMGR expression in *E. coli* (A) and activity assay of the purified enzyme. S1–S3 are three repetitions (B). The SDS-PAGE analysis indicated the expressed thHMGR was soluble in *E. coli* cytoplasm. lane 1, proteins having no affinity to Ni-NTA agarose resin were washed out at the first elution step; lane 2, total proteins obtained from cell lysis before Ni-NTA resin based purification treatment; lane 3 and 4, the thHMGR protein was washed out at the second elution step during Ni-NTA agarose resin based purification; M, protein marker. After purification, we assayed the activity of thHMGR through detecting the NADPH consumption. The decrease of absorbance at 340 nm was observed, indicating that the thHMGR expressed by *E. coli* retained the catalytic activity.

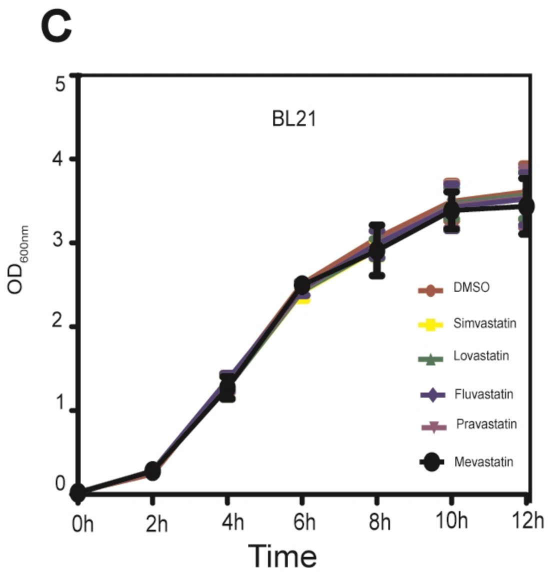

Figure S3. Toxicity test of statins on *E. coli* BL21. The toxicity testing experiments were performed with simvastatin, lovastatin, mevastatin, pravastatin, and fluvastatin. Results showed that none of them caused inhibition effect on growth of *E. coli* BL21. DMSO is the solvent of statins, which was also tested as a control.

Supporting Tables

Table S1. Docking results of ligands in BsFapR

**Ligand**	**Binding energy**	K_i
Malonyl-CoA	-4.34 kcal/mol	663.69 μM
HMG-CoA	-2.2 kcal/mol	24.28 mM

Table S2. Primers used in this study

Primers	Sequence
thHMGR-F	CGACGACGACAAGGCCATGGCTGATGCGGCGAAACACATC
thHMGR-R	CAGTGGTGGTGGTGGTGGTGCTCGATTAGAATTCTTCCGCGG
T7FapR-F	TAATACGACTCACTATAGGGAAAGAGGAGAAATAATGAGAA GAAATAAGAGAGAACG
T7FapR-R	CCGGACAATTAAGACTAGGTACTAATAGT TAAAGTTAAACAAA ATTAT
T7FapR-R2	CTCCTCTTTCCCTATAGTGAGTCGTATTACACATTCACCACCCTG AAT
fapO-F	ACTATTAGTACCTAGTCTTAATTGTCCGG ATAATTTTGTTTAAC TTTA
fapO-R	CTCCTCTTTCCCTATAGTGAGTCGTATTA CACATTCACCACCC TGAAT
PTrcfap-sfRfp-F	GTAGGGAACTGCCAGGCATC
PTrcfap-sfRfp-R	ATTCCGGTCGAGTGCCCACAC
pBad-F	ATCTGTGTGGGCACTCGACCGGAATCCATTCAGAGAAGAAACC
pBad-R	GTGAATAATTCTTCACCTTTAGACATATATAACCTCCTTAGAGCTC
pBad-F2	ATCTGTGTGGGCACTCGACCGGAAT
GFP-F	ATGTCTAAAGGTGAAGAA
GFP-R	TATTTGATGCCTGGCAGTTCCCTACTCACGCTGCAAGGGCGTAAT
GFP-R2	TATTTGATGCCTGGCAGTTCCCTAC
Trc-F	CAATTGTCTGATTCGTTACCAACGGTTCTGGCAAATATTCTG
Trc-R	CTGACGATGACACAATTTTTCATGGTTTATTCCTCCTT
33MevT-F	AAAAATTGTGTCATCGTCAG
33Mev-R	GGTAACGAATCAGACAATTG
33-F	GTCGACCTGCAGGCATGCAA
33-R	CATAGTGTAATCCTCCTTA
HMGR-F	ATAAGGAGGATTACACTATGGCGGCGAAACACATCCC
HMGR-R	TTGCATGCCTGCAGGTCGACTTAGAATTCTTCCGCGG
op1-Primer5	TTTTAAGAAGGAGATATACCACTATGTGTCAAATACCCCTAGAG
op1-Primer6	GGTATATCTCCTTCTTAAAAAATACCTGTAGGGGTATTT
pTet-Atcc-F	GATGTTAAAAAATAACTCGAGTAAGGATCTCCAGGCA
pTet-Atcc-R	TTATTTTTTAACATCGTAAGATCTTCTAAATTTGTCATCG
Trc-Forward	GTTTGACAGCTTATCATCGA
mkate-R	TCAACGATGTCCTAATTTCG
